# Selective Regional Chemotherapy of Unresectable Hepatic Tumours Using Lipiodol

**DOI:** 10.1155/1991/34537

**Published:** 1991

**Authors:** J. R. Novell, G. Dusheiko, N. I. Markham, K. Reddy, R. Dick, K. E. F. Hobbs

**Affiliations:** Hepatobiliary and Liver Transplantation Unit University Dept. of Surgery Royal Free Hospital School of Medicine Pond St London NW3 2QG UK

## Abstract

Over a 30 month period from 1987 to 1990, selective hepatic cannulation under fluoroscopic control was performed in 57 consecutive patients with primary and secondary malignancies of the liver. Fifty-three patients were subsequently treated using intra-arterial Lipiodol emulsified with epirubicin. The tumours treated were hepatocellular carcinoma (n = 35), metastatic adenocarcinoma (n = 14), intrahepatic cholangiocarcinoma (n = 3) and leiomyosarcoma (n = 1). For hepatocellular carcinoma the cumulative survival was 38% at one year; the median survival was 12.2 months for Stage I, 6.3 months for Stage II and 0.9 months for Stage III tumours.

In metastatic disease the cumulative survival was 63% at one year.

These data suggest that targeted intra-arterial chemotherapy with Lipiodol-epirubicin is a useful palliative therapy for patients with Stage and II HCC, and that a controlled trial of this treatment should be undertaken.


*HPB Surgery*, 1991, Vol. 4, pp. 223–236
Reprints available directly from the publisher
Photocopying permitted by license only

© 1991 Harwood Academic Publishers GmbH
Printed in the United Kingdom

### SELECTIVE REGIONAL CHEMOTHERAPY OF UNRESECTABLE HEPATIC TUMOURS USING LIPIODOL

J.R. NOVELL, G. DUSHEIKO, N.I. MARKHAM, K. REDDY, R. DICK and K.E.F. HOBBS

*Hepatobiliary and Liver Transplantation Unit, Royal Free Hospital School of Medicine, London, UK*

*(Received 6 November 1990)*

Over a 30 month period from 1987 to 1990, selective hepatic cannulation under fluoroscopic control was performed in 57 consecutive patients with primary and secondary malignancies of the liver. Fifty-three patients were subsequently treated using intra-arterial Lipiodol emulsified with epirubicin. The tumours treated were hepatocellular carcinoma (n = 35), metastatic adenocarcinoma (n = 14), intrahepatic cholangiocarcinoma (n = 3) and leiomyosarcoma (n = 1). For hepatocellular carcinoma the cumulative survival was 38% at one year; the median survival was 12.2 months for Stage I, 6.3 months for Stage II and 0.9 months for Stage III tumours.

In metastatic disease the cumulative survival was 63% at one year.

These data suggest that targeted intra-arterial chemotherapy with Lipiodol-epirubicin is a useful palliative therapy for patients with Stage I and II HCC, and that a controlled trial of this treatment should be undertaken.

KEY WORDS: Lipiodol, chemotherapy, liver tumours

### INTRODUCTION

Primary liver cancer or hepatocellular carcinoma (HCC) is one of the commonest and most malignant tumours in the world. It is especially prevalent in South-east Asia and Japan, where it ranks second only to stomach cancer, and in Sub-saharan Africa[1], where the incidence may be as high as 100 cases per 100,000 males per year[2]. Whilst the incidence is increasing in the United Kingdom[3], it remains far below that of secondary liver tumours which complicate one third of all extrahepatic malignant disease. This is particularly true for colorectal cancer, where 20% of patients have multiple liver metastases at the time of their initial surgery. Up to 50% of all patients who present with colorectal carcinoma will eventually develop liver deposits, but in many cases the liver is the only site of recurrent disease following a successful resection of the primary tumour[4]. The treatment of choice in both primary hepatocellular carcinoma and metastatic disease is surgical resection, but this is possible in less than a quarter of patients[1,4,5].

The prognosis is bleak in those patients with irresectable hepatic disease. Systemic chemotherapy and radiotherapy have a relatively low therapeutic index,

Address correspondence to: Mr J.R. Novell, MA, FRCS, University Dept. of Surgery, Royal Free Hospital School of Medicine, Pond St, London NW3 2QG, UK.

resulting in toxic side effects which are often unacceptable to the patient. This is partly a result of the high doses which are necessary to produce effective levels in tumour tissue.

Lipiodol Ultra-Fluid (May & Baker, England) is a lipid compound containing 475 mg of iodine per ml derived from poppyseed oil: it has been used for many years as a lymphographic contrast medium. When injected into the hepatic artery it is selectively retained in foci of hepatic tumour.In 1983 Konno *et al.* reported a reduction in tumour size and in concentrations of circulating tumour marker in 13 out of 14 patients with HCC following hepatic arterial infusion of Lipiodol mixed with the oily anticancer agent SMANCS (Styrene-maleic-anhydride-neocarzinostatin)[6]. A subsequent study of 124 oriental patients with primary and secondary liver tumours demonstrated significantly greater survival following infusion of a Lipiodol and SMANCS combination than hepatic artery ligation and chemotherapy[7]. Good response rates and increased survival have been reported using Lipiodol in combination with other cytotoxic agents including doxorubicin, mitomycin and cisplatinum[8,11]. The purpose of this study was to extend these initial findings by evaluating the efficacy and safety of Lipiodol-epirubicin therapy in a pilot group of patients with hepatic neoplasms.

### PATIENTS, MATERIALS AND METHODS

All patients with unresectable malignant tumours confined to the liver who were referred to this unit between November 1987 and February 1990 were offered Lipiodol-epirubicin treatment. Fifty-seven patients were entered into the study and fifty-three were treated. The treated tumours comprised thirty-five hepatocellular carcinomas, three intrahepatic cholangiocarcinomas, fourteen metastatic adenocarcinomas and one metastatic sarcoma. All had tumours which were initially considered unresectable, although hepatic resection was subsequently attempted in four patients. Surgery was contraindicated in multiple tumours involving both lobes of the liver, in the elderly, in patients with advanced cirrhosis in the surrounding liver parenchyma and in those with severely disordered hepatic function.

Four patients were excluded from treatment. In two anomalies of the hepatic artery prevented cannulation, and two patients were found to have total occlusion of the portal vein. No patients were excluded from Lipiodol-targeted therapy on the grounds of age, tumour size, impaired liver function or other possible adverse prognostic factors. The treated patients comprised 47 caucasians, 3 of oriental origin and 3 negroes. There were 39 men and 14 women, with a mean age of 58 years (range 34–81 years).

A histological diagnosis was obtained in 33 of 35 patients with hepatocellular carcinoma. In the two patients with a negative biopsy a hypervascular appearance at angiography in combination with a raised serum alpha fetoprotein (AFP) was regarded as diagnostic of HCC. In thirty-four patients the hepatocellular tumour occurred in a cirrhotic liver; one patient had a recurrence of a fibrolamellar tumour following previous resection. All hepatocellular tumours were staged according to the classification of Okuda[12] (Figure 1). Five patients had Stage 1 tumours at diagnosis (including two with newly-diagnosed recurrence following hepatic resection), 23 had Stage II malignancy, and 7 patients had Stage III tumours.

| | Tumour Size > 50% liver | Ascites present | Albumin < 30g/l | Bilirubin > 30 mg/l |
|---|---|---|---|---|
| Stage I | −ve | −ve | −ve | −ve |
| Stage II | | 1 or 2 +ve | | |
| Stage III | | 3 or 4 +ve | | |

**Figure 1** Staging of HCC (Okuda, 1985).

Of the 14 metastatic adenocarcinomas of gut origin, 10 were diagnosed at the time of resection of the primary tumour. Three patients developed metachronous hepatic lesions which were found on routine follow up, of whom two underwent partial hepatectomy with subsequent recurrence. The remaining patient presented with massive hepatomegaly: biopsy revealed a poorly differentiated adenocarcinoma but no primary tumour was identified. The known primary tumours comprised 12 carcinomas of colorectal origin and one gastric carcinoma. In the patient with metastatic leiomyosarcoma the diagnosis was made on liver biopsy.

All three patients with intrahepatic cholangiocarcinoma underwent percutaneous stent insertion for relief of obstructive jaundice prior to Lipiodol administration. Five patients (4 with colorectal metastases and one with cholangiocarcinoma) received systemic chemotherapy with 5FU and Mitomycin C in addition to Lipiodol-targeted treatment.

Intra-arterial Lipiodol-epirubicin injections were repeated at 2–3 monthly intervals in patients who showed no clinical deterioration. Serum levels of AFP and CEA were measured at diagnosis and before each treatment. Each patient received 40–120 mg of epirubicin (Pharmorubicin; Farmitalia, Italy), a water soluble cytotoxic agent which has been shown to possess equivalent activity against HCC and lower toxicity than its parent drug doxorubicin[13]. The dose for each patient was calculated on the basis of body surface area, liver function, and nutritional state (Table 1).

**Table 1** Formulation of Lipiodol-epirubicin emulsion.

**Epirubicin**75 $mg/m^2$.
Reduced by 25% if Bilirubin > 30 umol/l.
Reduced by 50% if Bilirubin > 100 umol/l.
**Lipiodol**10 ml.
**Urografin (Sodium meglumine diatrizoate)** 10 ml.

Since epirubicin is not lipid-soluble, the drug was dissolved in 10ml of 60% sodium meglumine diatrizoate (Urografin 290). This solution was then mixed for 5 mins with 10ml of Lipiodol using a Pulsatron ultrasonic agitator (Kerry Ultrasonics Ltd, England). The resulting stable colloidal emulsion was selectively infused into the hepatic artery via a catheter inserted into the femoral artery.

A CT scan of the liver was performed 10 days after each treatment when the Lipiodol had cleared from the non-neoplastic liver parenchyma. Deposits of HCC were typically hypervascular and showed a dense, even distribution of the lipid

(Figure 2), whereas uptake by metastases was less reliable and more commonly at the periphery of the tumour (Figure 3). The three patients with cholangiocarcinoma had characteristically hypovascular tumours and showed poor uptake of the Lipiodol emulsion.

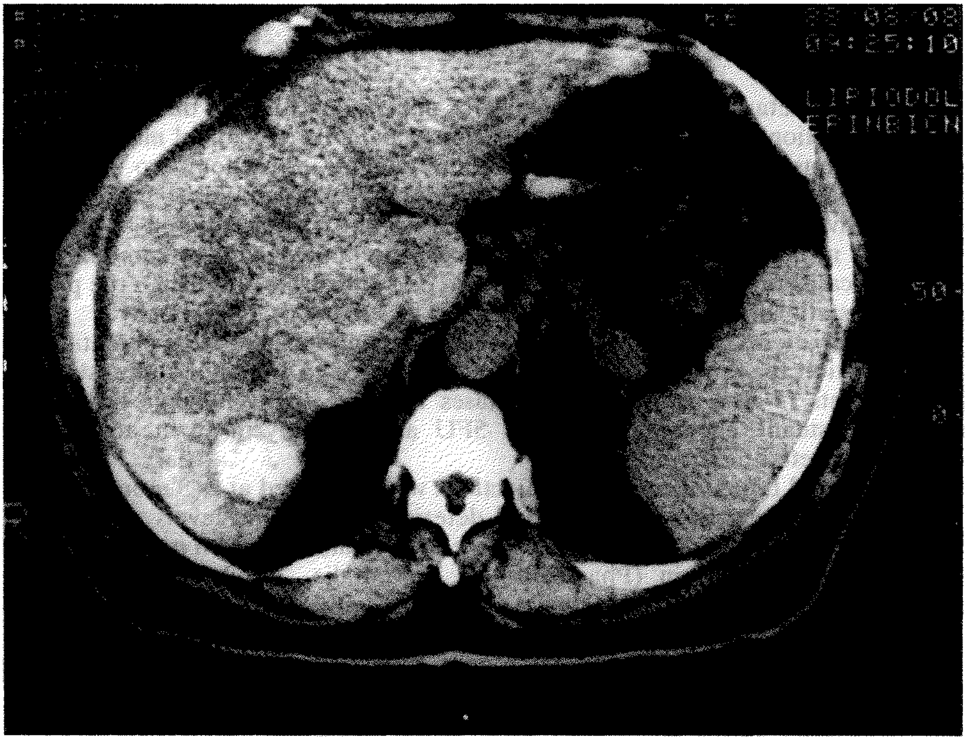

**Figure 2** Ct scan of liver showing dense uptake of Lipiodol in hepatocellular carcinoma.

### RESULTS

### *Survival*

#### *Hepatocellular carcinoma*

Four patients who underwent surgery within 2 months of their first treatment were excluded from analysis. Three of these had attempted resection of the tumour, all of whom succumbed to postoperative complications associated with their cirrhosis. One patient underwent liver transplantation and has subsequently died of recurrent disease.

Thirty one patients with HCC are evaluable: all have been followed for a minumum of 6 months from the start of treatment. Nineteen patients have died from progression of their tumour, and two from unrelated conditions. The cumulative survival at one year was 38% with a median survival of 6.0 months from

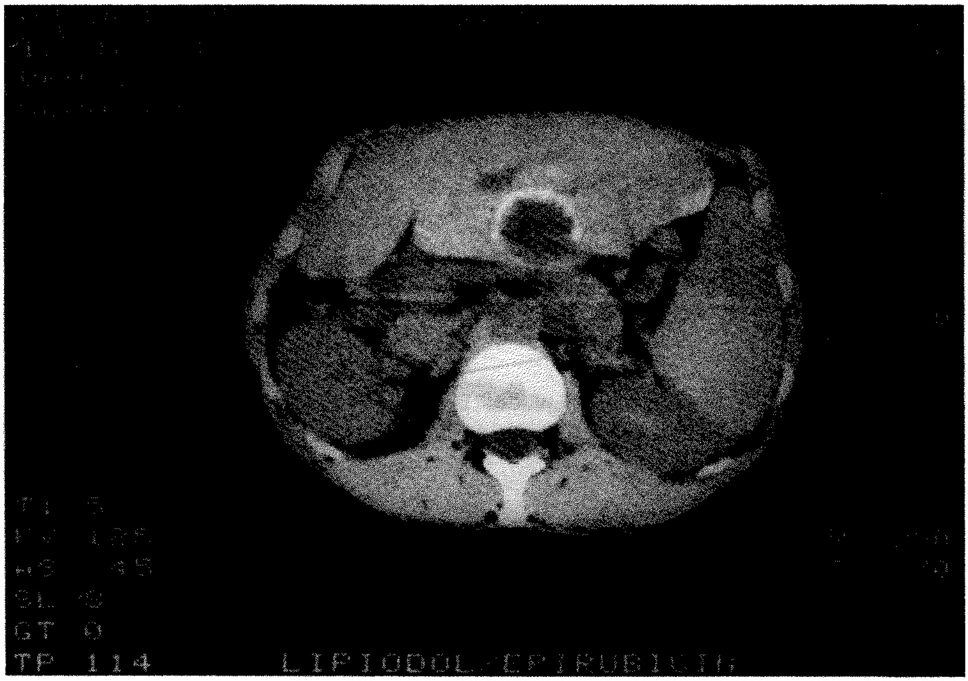

**Figure 3** CT scan of liver showing peripheral uptake of Lipiodol in metastatic adenocarcinoma.

diagnosis for all patients (range 0.5–27.0 months). The median survival by stages was as follows: Stage 1 tumours 12.2 months (range 6.5–14.8 months); Stage II tumours 6.3 months (range 1.3–27.0 months); Stage III tumours 0.9 months (range 0.5–1.5 months). The patient with recurrent fibrolamellar disease is alive and well 6 months following treatment. The cumulative survival of patients in each stage is summarised in Figure 4.

*Secondary tumours*

One patient underwent a successful resection of his tumour following the first treatment, and was excluded from analysis. Thirteen patients with metastatic adenocarcinoma are evaluable. Five patients are surviving between 6 and 13 months from the start of treatment, and eight died from tumour progression. The cumulative survival at 1 year was 63% (Figure 5), and the median survival was 11.0 months from diagnosis (range 0.5–27 months). Excluding those cases treated with systemic chemotherapy the median survival was 10 months (range 0.5–13 months). The patient with metastatic leiomyosarcoma is alive and well five months following Lipiodol-epirubicin treatment.

*Cholangiocarcinoma*

The three patients with intrahepatic cholangiocarcinoma died from tumour progression at 4.2, 12.3 and 20.9 months from diagnosis.

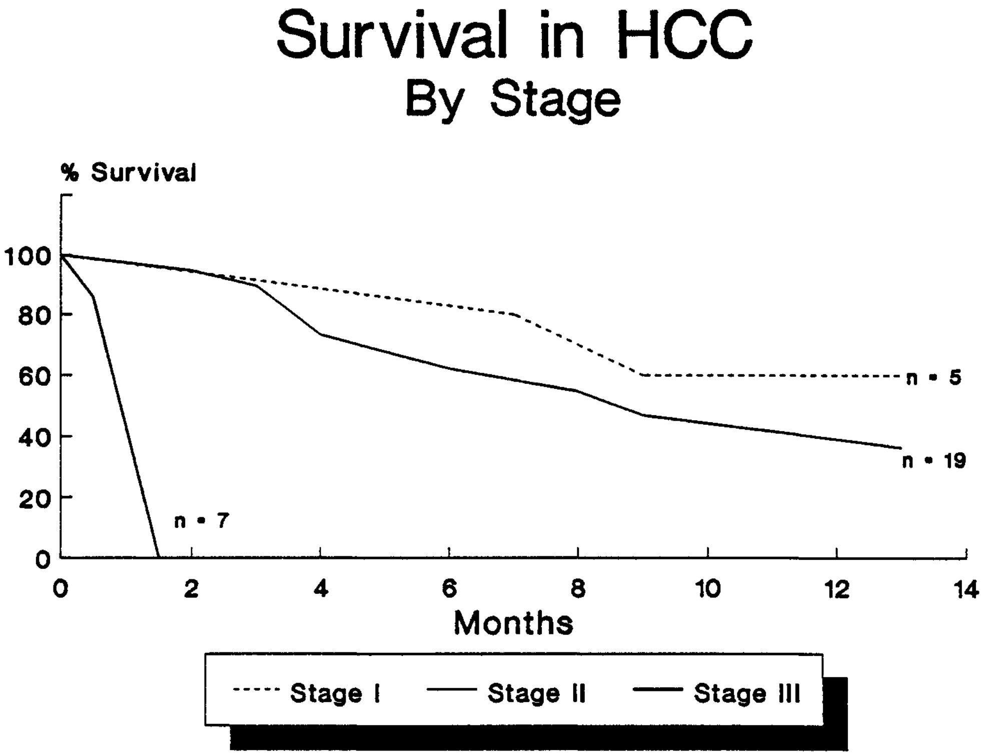

**Figure 4** Survival in HCC by stage.

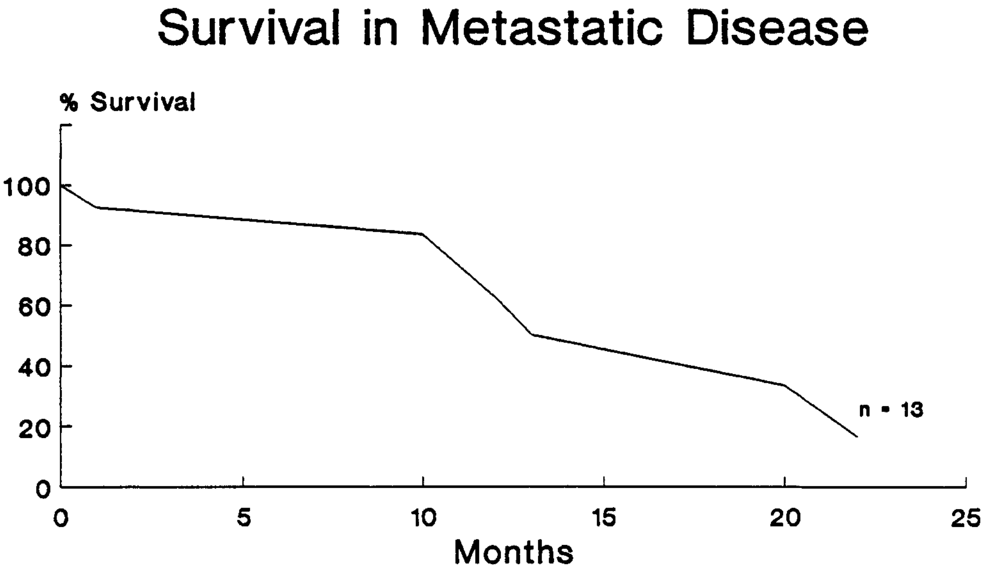

**Figure 5** Survival in metastatic disease.

*Inhibition of Tumour Growth*

Changes in tumour size were assessed on CT scans obtained 10 days following each treatment. Enlargement of hepatic tumour foci was evident in all three patients with cholangiocarcinoma and in 21 of the 37 patients (57%) with Stage I/II HCC and metastatic disease. Survival in Stage III HCC was too brief to allow reassessment of tumour size. In the remainder of patients with Stage I/II hepatocellular and metastatic tumours no increase in tumour volume was apparent on serial scans for periods of between 4 and 21 months (Figure 6). No consistent reduction in tumour size on serial CT scans was seen in any of the patients.

*Tumour Markers*

Prior to Lipiodol therapy the serum alpha fetoprotein level (AFP) was elevated in 22 patients with HCC (63%). Of these patients, only two showed a fall in AFP following treatment, and in one patient this was not sustained on subsequent treatments. One patient in whom the AFP was initially normal subsequently developed a raised serum level in association with progression of his disease after 11 months of treatment.

The serum CEA level was elevated in 9 patients with metastatic disease at diagnosis. Following the first treatment a fall occurred in two patients, one of whom maintained a normal level over 3 successive treatments; a further rise in CEA was associated with the development of lymphatic metastases.

*Technical Complications*

Four patients did not receive Lipiodol-targeted treatment due to vascular problems encountered at cannulation. In two, anomalies of the coeliac vascular tree prevented hepatic artery cannulation. The procedure was abandoned in two other patients who were found to have a total occlusion of the portal vein, because of the potentially lethal consequences of arterial occlusion. In one patient dissection of the hepatic artery occurred during cannulation: a successful treatment was administered 2 weeks later. Two patients developed an occlusion of the hepatic artery, each following 4 treatment episodes: further treatments were successfully administered via collaterals from the superior mesenteric artery. No haematoma, femoral artery thrombosis or false aneurysm occurred in any patient.

*Adverse Effects*

These were graded according to World Health Organisation criteria[14]. The most frequent side effects were pyrexia (37–39°C, Grade 1–2) which occurred in 83 treatments (74%), and elevation of serum transaminase levels in 75 treatments (67%). Abnormalities in liver function tests persisted for up to 10 days following therapy.

Three of seven patients with Stage III HCC died from treatment-related complications. Two patients with poor hepatic function developed fulminant liver failure following targeted chemotherapy, leading to death within 48 hours in both cases. The third patient developed Grade 4 leucopaenia and pneumonia and despite supportive therapy died 3 weeks later. There were no treatment-related deaths in patients with Stage I/II HCC, cholangiocarcinoma, or metastatic disease.

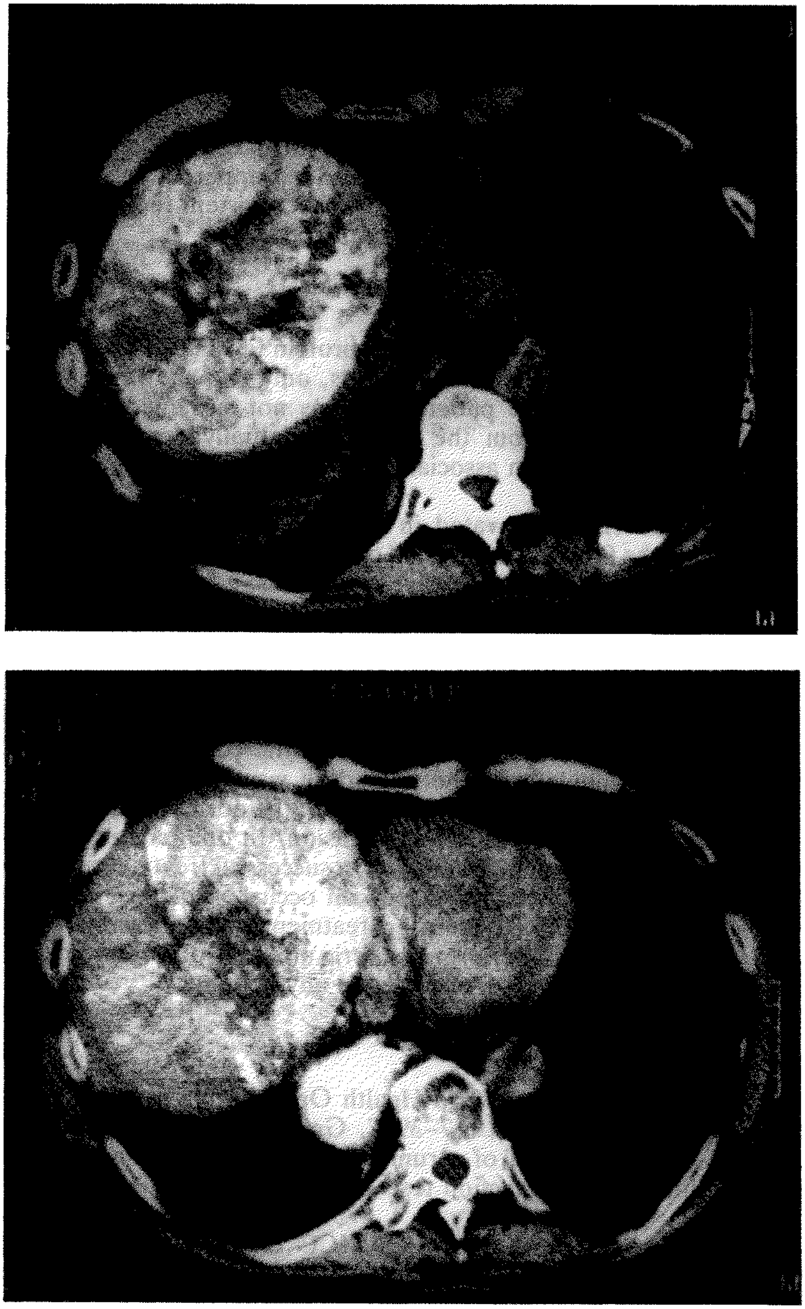

A

B

**Figure 6** A, B, C, D: Serial CT scans showing stasis in large hepatocellular carcinoma over a period of 18 months during treatment with Lipiodol-epirubicin.

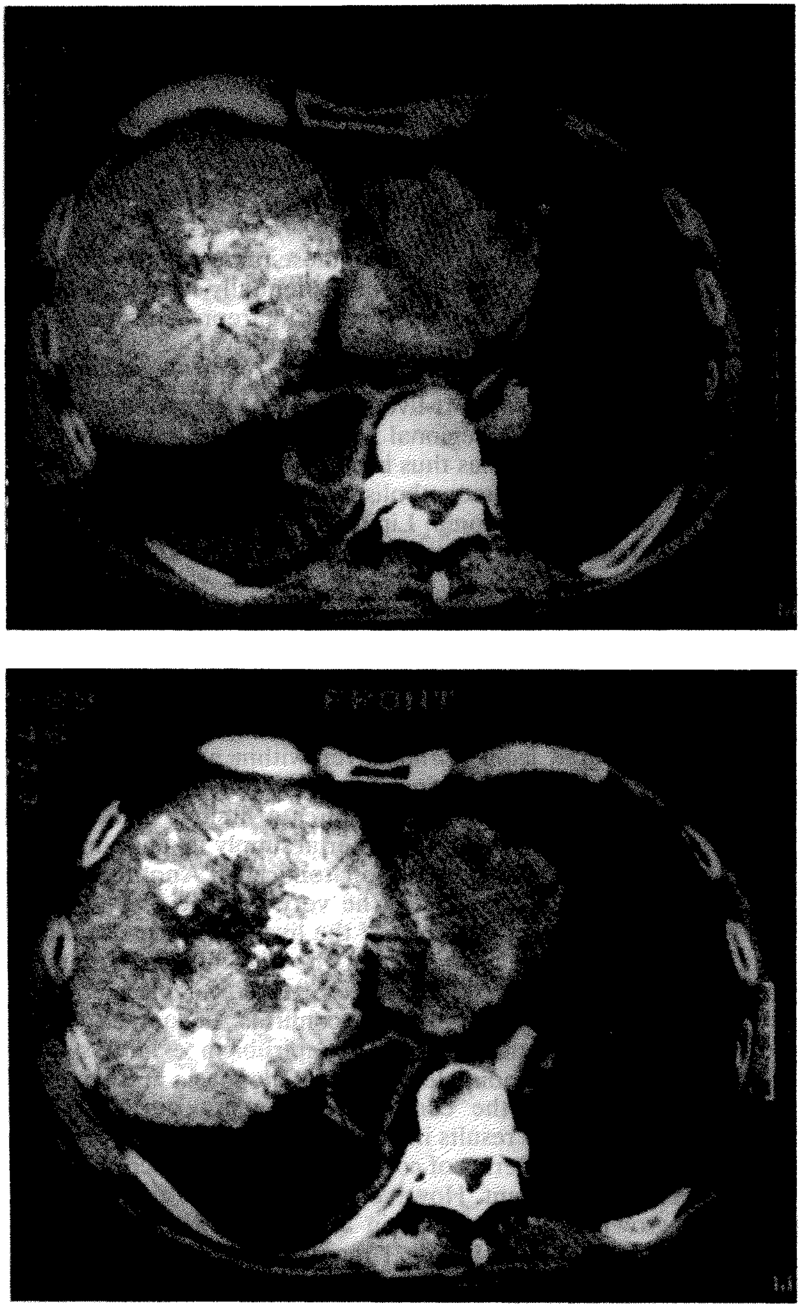

C

D

**Figure 6** continued

Other side effects were mild and transient. Mild to moderate abdominal pain occurred in patients (9%), and nausea and vomiting (Grade 1–2) in a further 8 cases (15%). Four patients (7%) developed partial, reversible alopecia (Grade 1–2) following treatment. No diarrhoea, mucositis or cardiac toxicity occurred in any patient.

### DISCUSSION

The prognosis in liver cancer remains poor, and these tumours are a major cause of mortality worldwide. Less than 25% of cases are potentially curable by surgery. For the majority of patients, therefore, improved palliation represents the only hope of prolonged survival.

Although up to one third of patients show a partial response to systemic chemotherapy, the benefit is often short-lived and there is no good evidence that it increases survival even if given by regional perfusion[15]. The possibility of specifically targeting therapy to tumours has thus been greeted with enthusiasm, particularly in Japan where Lipiodol "chemoembolisation" is now the first line treatment for inoperable hepatocellular carcinoma. Lipiodol-targeted chemotherapy has also been employed in tumours of the lung, gallbladder and pancreas[16] and of the bladder[17].

The precise mechanisms of Lipiodol retention remain speculative. In particular, there is much debate as to whether this is an embolic phenomenon, with the Lipiodol being trapped in the microvasculature of the tumour circulation, or uptake by a population of tumour cells. *In vivo* microscopy of the liver following Lipiodol injection has shown active uptake of the lipid by cells of the reticulo-endothelial system[18]. The lipid is cleared from normal liver within a few days, possibly by shunting into the hepatic veins via the sinusoids.

The technique of "chemoembolisation" requires a limited number of feeding vessels which constitute the sole or principal arterial supply to a tumour, and in this respect the liver is particularly well suited anatomically. Another advantage of targeted chemotherapy in liver tumours is the very low incidence of associated toxicity. This is partly due to the efficiency of the liver macrophages in "scavenging" oily emulsions[18], and partly a function of the high first-pass metabolism of cytotoxics such as epirubicin[19]. These factors result in lower plasma levels of the drug and few systemic side effects, as this study confirms. In contrast, over 50% of patients receiving systemic chemotherapy with epirubicin exhibit symptoms of immediate and early toxicity including leucopaenia, alopaecia and vomiting[20].

Despite previous reports to the contrary[8,9], it is not our experience that Lipiodol-targeted chemotherapy results in either tumour shrinkage or reduction in levels of circulating markers. In terms of conventional response criteria, therefore, no response was seen in any patient. However, in about half of the cases studied a plateau in tumour growth was observed, in association with continued clinical wellbeing, for periods of between 4 and 21 months. No data is available on the stability of the Lipiodol-epirubicin emulsion within tissues, but it seems reasonable to postulate that the Lipiodol-epirubicin emulsion acts as a slow release preparation, inhibiting local tumour growth. Alternatively, tumour necrosis resulting from the treatment may not be evident on hepatic imaging. In theory the use of a more effective cytotoxic "warhead" such as the isotope Iodine-131 would result in greater tumour destruction. Such a vehicle is now available and early experience from clinical trials has been encouraging, with reduction in tumour size and ascites and symptomatic improvement in the majority of patients[21,22]. We are currently investigating $^{131}$I-Lipiodol as a treatment for both HCC and cholangiocarcinoma.

This was an uncontrolled study, and it is therefore unwise to be too dogmatic about the efficacy of Lipiodol-targeted chemotherapy. However, the overall survival of patients with Stage I and II HCC in this study is better than previously published rates for patients receiving systemic chemotherapy[13] and for untreated patients[12], in whom the prognosis is uniformly poor. Results in Stage III tumours remains dismal with a median survival of less than one month.

In conclusion, our experience with Lipiodol-targeted chemotherapy in nearly 60 patients suggests that in patients with Stage I and II hepatocellular carcinoma it is a safe technique with a low complication rate. Systemic side effects are less frequent and less severe than those reported with intravenous chemotherapy. No significant reductions in tumour volumes were observed during treatment, but survival in these patients was promising. Randomised controlled trials of Lipiodol-targeted therapy are indicated in patients with Stage I and II tumours. The technique appears of no value in the palliation of Stage III HCC and carries a significant mortality in this group. Likewise, in the small number of patients with cholangiocarcinoma or metastatic tumours in this series, no convincing benefit was seen.

*Acknowledgements*

The authors wish to thank the staff of the Royal Free Hospital Pharmacy Sterile Laboratory for preparing the Lipiodol-epirubicin emulsion used in this study.

This work has been carried out as part of the requirement for the degree of MChir of the University of Cambridge.

*References*

1. Takayasu, K. Shima, Y. Muramatsu, Y. *et al.* (1987) Hepatocellular carcinoma: treatment with intra-arterial iodized oil with and without chemotherapeutic agents. *Radiology*, **162**, 345–351
2. Sherlock, S. (1989) *Diseases of the liver and biliary system*. 8th edition. Oxford: Blackwell
3. Burnett, R.A., Patrick, R.S., Spilg, W.C.S. *et al.* (1987) Hepatocellular carcinoma and hepatic cirrhosis in the west of Scotland: a 25 year necropsy review. *J. Clin. Pathol.*, **31**, 108–110
4. Taylor, I. (1981) Studies on the treatment and prevention of colorectal liver metastases. *Ann. R. Coll. Surg.*, **63**, 270–276
5. Maraj, R. Kew, M.C., Hyslop, R.J. (1988) Resectability rate of hepatocellular carcinoma in rural southern Africans. *Br. J. Surg.*, **75**, 335–338
6. Konno, T., Maeda, H., Iwai, K. *et al.* (1983) Effect of arterial administration of high-molecular-weight anticancer agent SMANCS with lipid lymphographic agent on hepatoma: a preliminary report. *Eur. J. Cancer Clin. Oncol.*, **19**, 1053–1065
7. Tashiro, S., Maeda, H. (1985) Clinical evaluation of arterial administration of SMANCS in oily contrast medium for liver cancer. *Jap. J. Med.*, **24**, 79–80
8. Kanematsu, T., Furuta, T., Takenaka, K. *et al.* (1989) A 5 year experience of lipiodolization: selective regional chemotherapy for 200 patients with hepatocellular carcinoma. *Hepatology*, **10**, 98–102
9. Kobayashi, H., Shimida, H., Yano, T. *et al.* (1987) Intra-arterial injection of adriamycin/mitomycin C Lipiodol suspension in liver metastases. *Acta Radiolog.*, **28**, 275–280
10. Ohishi, H., Uchida, H., Yoshimura, H. *et al.* (!985) Hepatocellular carcinoma detected by iodized oil; use of anticancer agents. *Radiology*, **154**, 25–29

11. Chin, J.H., Oon, C.J., Tan, L. and Yong, Y.M. (1986) Treatment of irresectable hepatocellular carcinoma with intrahepatic arterial Lipiodol mixed with adriamycin and mitomycin C. *Ann. Acad. Med. Singapore,* **15**, 162–168
12. Okuda, K., Ohtsuki, T., Obata, H., Tomimatsu, M., Okazaki, N., Hasegawa, H., Nakajima, Y. and Ohnishi, K. (1985) Natural history of hepatocellular carcinoma and prognosis in relation to treatment. *Cancer* **56**, 918–928
13. Hochster, H.S., Green, M.D., Speyer, J. *et al.* (1985) 4'epidoxorubicin (Epirubicin): activity in hepatocellular carcinoma. *J. Clin. Oncol.*, **3**, 1535–1540
14. WHO handbook for reporting results of cancer treatment (1979) Geneva: World Health Organisation
15. Malik, S.T.A. and Wrigley, P.F.M. (1988) Intra-arterial hepatic chemotherapy for liver malignancy. *Br. Med. J.*, **297**, 434
16. Konno, T. *et al.* (1984) Selective targeting of anti-cancer drug and simultaneous image enhancement in solid tumours by arterially administered lipid contrast medium. *Cancer,* **54**, 2367–2374
17. Ozono, S., Okajima, E., Hirao, Y., Babaya, K., Komada, S., Matsuki, H., Takahashi, S., Ohishi, H., and Yoshioka, T. (1988) Transcatheter arterial embolization of vesical artery in the treatment of invasive bladder cancer. *Eur. Urol.*, **15**(3–4), 176–9
18. Ivancev, K., Lunderquist, A., McCuskey, R., McCuskey, P. and Wretlind, A. (1989) Effect of intravenously injected iodinated lipid emulsions on the liver. *Acta Radiol.*, **30**, 291–298
19. Strocchi, E., Camaggi, C.M., Rossi, A.P. *et al.* Epirubicin pharmacokinetics after intrahepatic arterial and intraperitoneal administration. *Drugs Exptl. Clin. Res.*, **XI**, 295–301
20. Ganzina, F., Di Pietro, N. and Magni, O. Clinical Toxicity of 4'-epi-doxorubicin (epirubicin). *Tumori*, **71**, 233–240
21. Park, C.H., Suh, J.H., Yoo, H.S. *et al.* (1987) Treatment of hepatocellular carcinoma (HCC) with radiolabelled Lipiodol: a preliminary report. *Nucl. Med. Commun.*, **8**, 1075–1087
22. Bretagne, J, Raoul, J., Bourguet, P. *et al.* (1988) Hepatic artery injection of I-131-labelled Lipiodol. Part II; preliminary results of therapeutic use in patients with hepatocellular carcinoma and liver metastases. *Radiology*, **168**, 547–550

*(Accepted by S. Bengmark 6 January 1991)*

### INVITED COMMENTARY

Both transcatheter arterial embolization and intraarterial infusion chemotherapy have been utilized to treat patients with regional tumors at M.D. Anderson Cancer Center. In primary and metastatic hepatic neoplasms both arterial tumors embolization and infusion chemotherapy showed an improved tumor response rate when compared with traditional systemic chemotherapy. In a group of patients with hepatic metastases from colon carcinoma who failed systemic chemotherapy, intraarterial infusion chemotherapy with the same drugs yielded a 43% response rate[1].

The combination of arterial embolization, infusion chemotherapy, and chemoembolization has also been attempted to achieve maximal tumor response. Chemoembolization theoretically, results in ischemia, increased capillary permeability and an increased contact time between the cytotoxic agent and the tumor cells. This has been performed in several fashions: (1) embolization of one lobe and infusion of the non-embolized lobe, (2) sequential embolization and infusion of the same lobe, (3) chemoembolization using chemotherapeutic drugs mixed with arterial occlusive particulates, (4) microcapsule embolization and (5) chemoembolization using oily substance as a carrier for chemotherapeutic drugs. Chemoembolization using Cisplatin mixed with Gelfoam powder or segments (Upjohn Inc., Kalamazoo, MI) or Ivalon particles (Polyvinyl alcohol foam,

Unipoint Laboratories, High Point, NC) has resulted in an improved tumor response in patients with metastatic ocular melanoma to liver and pelvic tumors in our institution[2]. Chemoembolization using microcapsules or starch particles has also been attempted to combine the effect of embolization and chemotherapy in various tumors[3,4].

The use of an oily substance such as Lipiodol Ultrafluid (Ethiodol), the ethylester of poppyseed oil containing Iodine, as a drug carrier represents another form of chemoembolization. It has been shown in operative specimens that Lipiodol does retain the lipid soluble Adriamycin, however this may not be valid for water soluble cytotoxic agents. Hypervascular neoplasms are more responsive. Preliminary reports from several centers of chemoembolization using Lipiodol-drug mixture have demonstrated improved tumor response with a reduction of tumor size and vascularity[5–8]. In the current study by Novell *et al.*, using Lipiodol-epirubicin mixture for chemoembolization, arrest of tumor progression was observed in half of the patients, but none had a significant reduction in tumor size. The discrepancy of the tumor response rate between this and other Japanese series suggests that epirubicin as the sole agent for Lipiodol chemoembolization was less effective than other agents such as Adriamycin, Mitomycin, Cisplatin, SMANCS, or other drugs in combinations. The addition of other chemotherapeutic drugs into the Lipiodol-epirubicin combination would appear to be warranted in their future study.

*In vivo* microscopy of the rat liver after Lipiodol injection into the hepatic artery by Kan *et al.*, showed that large amounts of Lipiodol entered the portal vein through arterioportal shunting, then reached the hepatic sinusoids and eventually exited from the hepatic veins[9]. In over thirty patients who received chemoembolization using a mixture of Ethiodol, multiple drugs and arterial blocking agents (Ivalon particles; and Angiostat, Regional Therapeutics Inc., Santa Monica, CA), we have observed portal vein opacification by Ethiodol in two patients, thus confirming the presence of arterioportal shunting in the human liver.

Although the microcirculation of human liver might not be exactly similar to that of the rat liver, the presence of arterioportal shunting is alike. Chemoembolization of human liver using oily substance results in combined arterial and portal embolization, or total hepatic parenchymal and tumoral embolization. However, due to fast clearance of Lipiodol from the normal sinusoids within several hours to days and prolonged existence of Lipiodol within the tumor for weeks to months, most patients have tolerated Lipiodol chemoembolization without significantly higher complications than those who received other embolization methods. Because of the total hepatic embolization nature of Lipiodol chemoembolization intentional segmental infarction of the liver had been achieved by this method[10]. It is imperative that for palliative control of hepatic neoplasm not be excessive and only segmental or lobar hepatic embolization, instead of total hepatic embolization, is performed at one setting.

### *References*

1. Patt, Y., Peter, R.E., Chuang, V.P., Wallace, S., and Mavligit, G.M. (1983) Effective pretreatment of colorectal and liver metastases. *Am. J. Med.*, **75**, 237–240
2. Mavligit, G.M., Charnsangavej, C., Carrasco, C.H., Patt, Y.Z., Benjamin, R.S., and Wallace, S. (1988) Regression of ocular melanoma metastatic to the liver after hepatic artery chemoembolization with cisplatin and polyvinyl sponge. *JAMA*, **260**(7), 974–976

3. Kato, T., Nemoto, R., Mori, H., Takahaski, M., Tamakawa, Y., and Harada, M. (1981) Arterial chemoembolization with microencapsulated anticancer drug. *JAMA*, **245**, 1123–1127
4. Ensminger, W.D., Dakhill, S., and Cho, K. (1980) Improved regional selectivity of hepatic arterial bischlorethylnitrosourea plus degradable starch microspheres (abstracted). *Clin. Res.*, **28**, 742
5. Konno, T., Maeda, H., Iwai, K., *et al.* (1983) Effect of arterial administration of high-molecular-weight anticancer agent SMANCS with lipid lymphographic agent on hepatoma: a preliminary report. *Eur. J. Cancer Clin. Oncol.*, **19**, 1053–1065
6. Kanematsu, T., Furuta, T., Takenaka, K., *et al.* (1989) A 5 year experience of lipiodolization: selective regional chemotherapy for 200 patients with hepatocellular carcinoma. *Hepatology*, **10**, 98–102
7. Kobayashi, H., Shimida, H., Yano, T., *et al.* (1987) Intra-arterial injection of adriamycin/mitocyin C Lipiodol suspension in liver metastases. *Acta Radiol.*, **28**, 275–280
8. Ohishi, H., Uchida, H., Yoshimura, H. *et al.* (1985) Hepatocellular carcinoma detected by iodized oil: use of anticancer agents. *Radiology*, **154**, 25–29
9. Zan, Z., Ivancev, K., Hagerstrand, I., Chuang, V.P. and Lunderquist, A. (1989) *In vivo* microscopy of the liver after injection of lipiodol into the hepatic artery and portal vein in the rat. *Acta Radiol.*, **30**, 419–425
10. Nakamura, H., Hashimoto, T., Oi, H., Sawada, S., Furui, S., and Mizumoto, S. (1990) Treatment of hepatocellular carcinoma by segmental hepatic artery injection of adriamycin-in-oil emulsion with overflow to segmental portal veins. *Acta Radiol.*, **31**(4), 347–349

Vincent P. Chuang
Sidney Wallace
Univ. of Texas
Houston, USA

